# Coordination of Tetracyanoquinodimethane-Derivatives with Tris(pentafluorophenyl)borane Provides Stronger p-Dopants with Enhanced Stability

**DOI:** 10.1021/acsami.3c10373

**Published:** 2023-09-20

**Authors:** Ahmed E. Mansour, Ross Warren, Dominique Lungwitz, Michael Forster, Ullrich Scherf, Andreas Opitz, Moritz Malischewski, Norbert Koch

**Affiliations:** † Helmholtz-Zentrum Berlin für Materialien und Energie GmbH, 12489 Berlin, Germany; ‡ Institut für Physik & IRIS Adlershof, Humboldt-Universität zu Berlin, 12489 Berlin, Germany; § Department of Chemistry and Wuppertal Center for Smart Materials and Systems (CM@S), Bergische Universität Wuppertal, 42097 Wuppertal, Germany; ∥ Institute of Chemistry and Biochemistry, Freie Universität Berlin, 14195 Berlin, Germany

**Keywords:** organic semiconductor, doping, dopants, thin films, charge transport

## Abstract

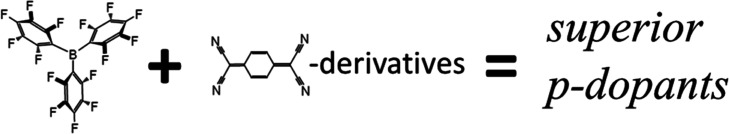

Strong molecular dopants for organic semiconductors that are stable against diffusion are in demand, enhancing the performance of organic optoelectronic devices. The conventionally used p-dopants based on 7,7,8,8-tetracyanoquinodimethane (TCNQ) and its derivatives “F*x*TCN(N)Q”, such as 2,3,4,6-tetrafluoro-7,7,8,8-tetracyanoquinodimethane (F4TCNQ) and 1,3,4,5,7,8-hexafluorotetracyano-naphthoquinodimethane (F6TCNNQ), feature limited oxidation strength, especially for modern polymer semiconductors with high ionization energy (IE). These small molecular dopants also exhibit pronounced diffusion in the polymer hosts. Here, we demonstrate a facile approach to increase the oxidation strength of F*x*TCN(N)Q by coordination with four tris(pentafluorophenyl)borane (BCF) molecules using a single-step solution mixing process, resulting in bulky dopant complexes “F*x*TCN(N)Q-4(BCF)”. Using a series of polymer semiconductors with IE up to 5.9 eV, we show by optical absorption spectroscopy of solutions and thin films that the efficiency of doping using F*x*TCN(N)Q-4(BCF) is significantly higher compared to that using F*x*TCN(N)Q or BCF alone. Electrical transport measurements with the prototypical poly(3-hexylthiophene-2,5-diyl) (P3HT) confirm the higher doping efficiency of F4TCNQ-4(BCF) compared to F4TCNQ. Additionally, the bulkier structure of F4TCNQ-4(BCF) is shown to result in higher stability against drift in P3HT under an applied electric field as compared to F4TCNQ. The simple approach of solution-mixing of readily accessible molecules thus offers access to enhanced molecular p-dopants for the community.

## Introduction

Molecular doping of organic semiconductors (OSCs) is a key strategy to enhance the performance of organic optoelectronic devices by increasing the density of mobile charge carriers in the OSC and tuning the energy barriers at interfaces between the various layers in devices.^1–4^ In p-doping of OSCs, molecular oxidants (p-dopants) are introduced into the matrix of a polymeric or molecular semiconductor (host) to create additional charge carriers in the host material, thereby increasing its electrical conductivity and shifting the Fermi level toward the valence band of the polymer host or toward the highest occupied molecular orbital (HOMO) level in molecular hosts.^4–6^

Over the past years, several p-doping mechanisms have been identified and investigated, such as integer charge transfer (ICT) to the dopant molecule, the use of charge-transfer complexes as dopants, and more recently *via* the protonation of polymer chains by Lewis acids with subsequent charge transfer to a neutral chain segment.^5,7–9^ Among the various doping mechanisms, using conjugated molecules with a strong oxidation potential to undergo ICT upon mixing with OSCs has been a traditional and universal approach capable of achieving high doping efficiency—defined as the number of free charge carriers produced by each introduced dopant molecule.^2,5^

In a simplified picture of ICT, the transfer of a single electron from the host OSC to the dopant molecule requires that the electron affinity (EA) of the dopant be higher than (or at least in close proximity to) the ionization energy (IE) of the host OSC so that an electron transfer from the highest occupied electronic level (valence band or HOMO) of the host to the lowest unoccupied molecular orbital level of the molecular dopant is energetically feasible. Accordingly, a strong p-dopant is one that possesses a high EA compared to the IE of the OSC.^4,5,10,11^ The increasing number of high-performing OSCs with rather high ionization energy (IE > 5.4 eV) has pushed the development of strong p-dopants with higher EA.^2,12–15^

7,7,8,8-Tetracyanoquinodimethane (TCNQ) is an electron acceptor with an EA in the range of 3.38 to 4.23 eV^16,17^ and has received vast research interest due to its demonstrated success in forming highly conductive charge-transfer salts.^18–20^ Fluorination of TCNQ, in which the hydrogen atoms at the quinoid ring are replaced by fluorine atoms, enables the synthesis of further electron-accepting molecules F*x*TCNQ (*x* = 1, 2, 3, and 4) with higher EA, depending on the number of added fluorines.^17^ The most popular of these is 2,3,4,6-tetrafluoro-7,7,8,8-tetracyanoquinodimethane (F4TCNQ), which has been extensively used as a p-dopant with reported EA values in the range of 5.08 to 5.33 eV.^6,17,21,22^ A related dopant with even higher EA, 1,3,4,5,7,8-hexafluorotetracyano-naphthoquinodimethan (F6TCNNQ) (in the range of 5.00 to 5.60 eV), has also been demonstrated as a strong p-dopant for polymer semiconductors.^12,23–26^ However, the TCNQ family of p-dopants “F*x*TCN(N)Q (*x* = 0, 1, 2, 3, 4, or 6)” were observed to exhibit poor thermal stability in OSCs as well as significant electric field-induced diffusion under device operation conditions.^21,22,27–29^ Furthermore, their 2D planar structure allows for the possibility of forming charge-transfer complexes due to overlap between the frontier orbitals of the dopant molecules and host material, which results in lower doping efficiency compared to the ICT case.^5,30–32^ Accordingly, the research community has been motivated to develop stronger p-dopants (exhibiting higher EA) with a bulkier structure to enhance doping efficiency and dopant stability against diffusion. Examples include C_60_F_36_ (EA ≈ 5.38 eV),^27,33^ Mo(tfd)_3_ and its soluble derivatives (EA in the range 5.00 to 5.60 eV),^7,34–37^ CN6-CP (EA ≈ 5.87 eV),^12,14^ and Mes_2_B^+^[B(C_6_F_5_)_4_]^−^ (EA_eff_ ≈ 5.90 eV).^15,38^

Recently, the reduction potential of TCNQ was reported to increase strongly when being combined with 4 equiv of tris(pentafluorophenyl)borane [B(C_6_F_5_)_3_, commonly abbreviated as BCF]. BCF is a strong Lewis acid which increases the electron deficiency of TCNQ upon coordination, leading to an increase in oxidation power. Upon reduction, four BCF molecules can coordinate with all four CN groups of TCNQ^–^, leading to a better charge delocalization in [TCNQ·4BCF]^−^, which explains its possible coexistence in the presence of strongly oxidizing cations.^39^ We hypothesized that extending this process to stronger TCNQ derivatives might offer the opportunity for a facile method to produce stronger molecular p-dopants. In this work, we validate this hypothesis for TCNQ, F4TCNQ, and F6TCNNQ, and we present a simple single-step solution mixing process of commercially available molecules, namely BCF with F*x*TCN(N)Q (*x* = 0, 4, and 6), to produce bulkier molecular dopants “F*x*TCN(N)Q-4(BCF)” with a demonstrated increase in the oxidation strength. The increased oxidation strength of the BCF coordinated dopants is demonstrated to enable effective p-doping of polymers with rather high IE, namely methylated poly(*para*-phenylene) (MeLPPP, IE ≈ 5.40 eV)^15^ and poly(9,9-dioctylfluorene-*alt*-benzothiadiazole) (F8BT, IE ≈ 5.90 eV),^15^ in contrast to F*x*TCN(N)Q or BCF alone as dopants, which achieve only a negligible degree of doping. We also show that the degree of doping and the increase in the electrical conductivity for the prototypical poly(3-hexylthiophene-2,5-diyl) (P3HT, IE ≈ 4.70 eV)^15^ are higher when doped with BCF-coordinated F4TCNQ as compared to doping with F4TCNQ or BCF alone. Furthermore, the dopant stability against drift under an applied electrical field and the thermal stability of the doped P3HT film are enhanced for F4TCNQ when coordinated with BCF due to the bulkier and larger size of that dopant.

## Results and Discussion

**Figure 1:**
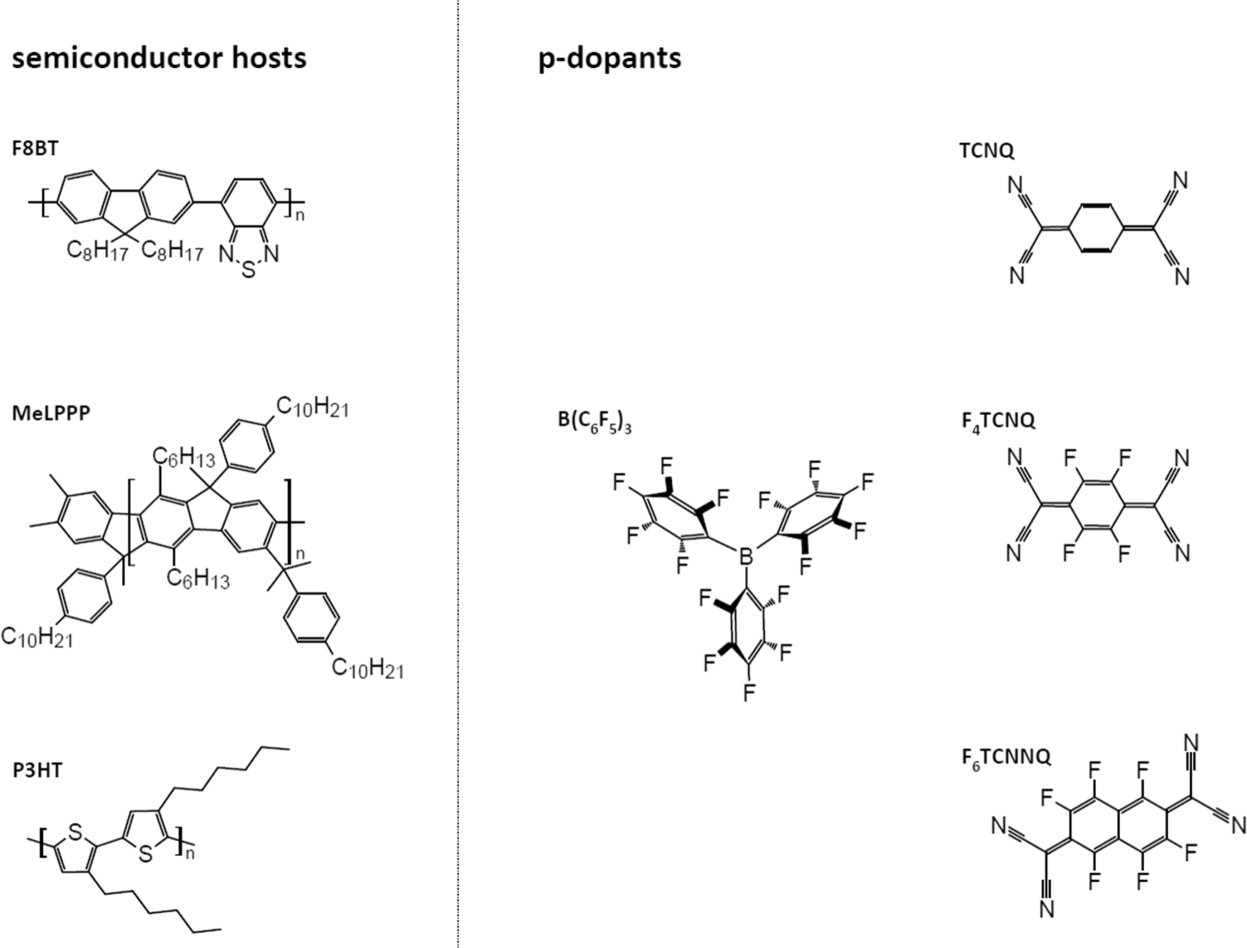
Chemical structure of the molecular dopants and polymer semiconductor hosts used in this work.

The chemical structures of the molecular dopants and the host polymers used in this work are shown in Figure 1. We first demonstrate the effect of BCF coordination on F*x*TCN(N)Q on the oxidation strength by comparing the pairs (dopant/host): TCNQ:P3HT, F4TCNQ:MeLPPP, and F6TCNNQ:F8BT, to those including the BCF coordinated dopants, namely TCNQ-4(BCF):P3HT, F4TCNQ-4(BCF):MeLPPP, and F6TCNNQ-4(BCF):F8BT. The selection of the host polymers was based on their increasing IE (P3HT < MeLPPP < F8BT). This is an effective approach used to evaluate the strength of new dopants by attempting to dope polymer hosts with progressively increasing IE.^15^ The dopant and host combinations were selected to create a valid comparison between the case in which F*x*TCN(N)Q alone does not dope (or weakly dope) the host and the case in which coordinated F*x*TCN(N)Q dopes the same host material. More specifically, the initial choice of the F*x*TCN(N)Q dopant was based on the offsets between the IE of the host and the EA of the dopant, which are not expected to lead to ICT.

Then, we compare the performance of F4TCNQ:P3HT to that of F4TCNQ-4(BCF):P3HT in terms of electrical conductivity and dopant stability in the host under an applied electrical field and thermal treatment.

**Figure 2:**
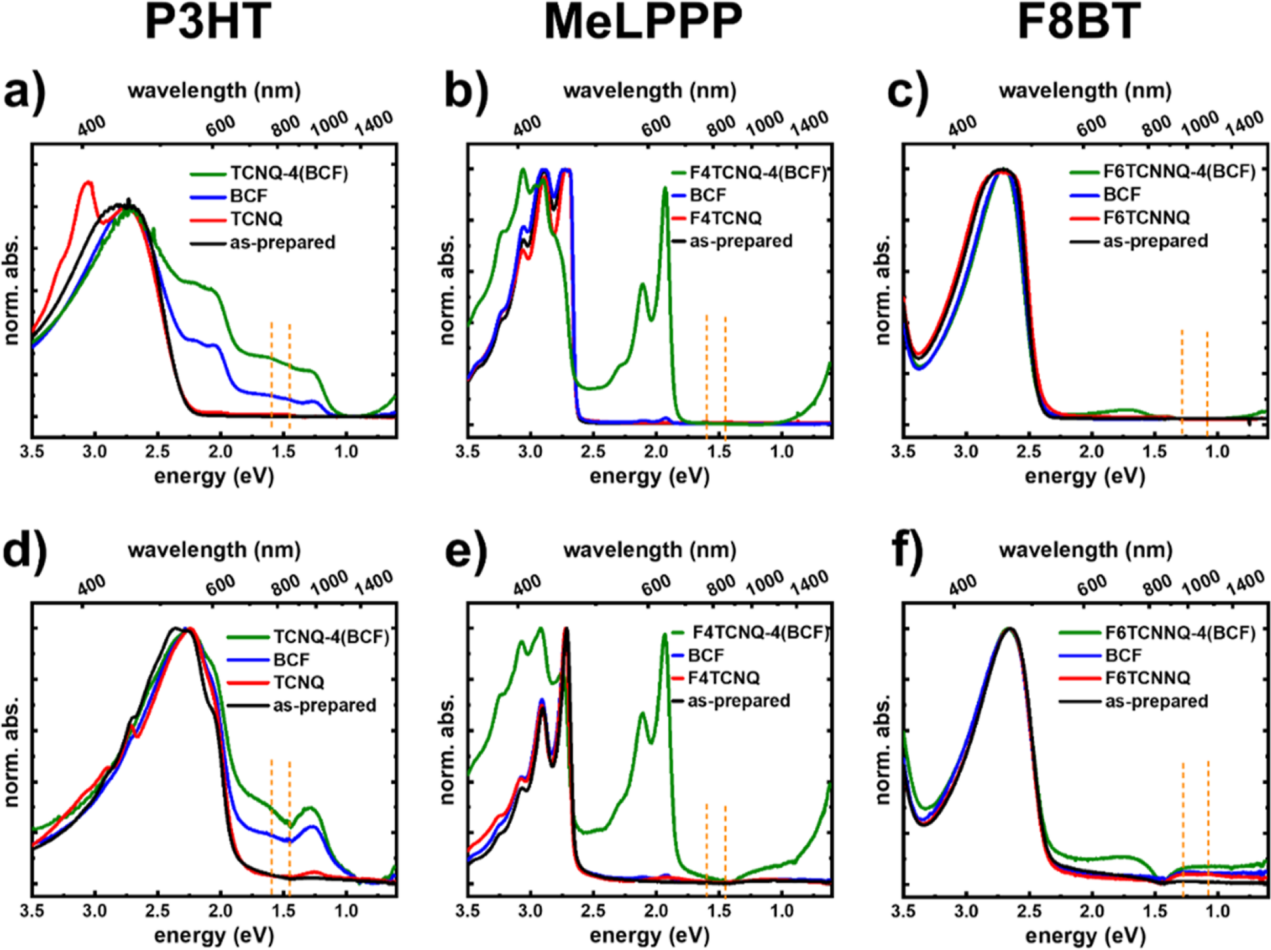
Optical absorption spectra of the as-prepared polymers and their changes upon doping with different dopant molecules. Top panels show the spectra measured in solution for (a) as-prepared P3HT, TCNQ:P3HT (1:10), BCF:P3HT (4:10), and TCNQ-4(BCF):P3HT (1:30) in CHCl_3_, (b) as-prepared MeLPPP, F4TCNQ:MeLPPP (1:10), BCF:MeLPPP (4:10), and F4TCNQ-4(BCF):MeLPPP (1:10) in *o*-DCB, and (c) as-prepared F8BT, F6TCNNQ:F8BT (1:10), BCF:F8BT (4:10), and F6TCNNQ-4(BCF):F8BT (1:10) in *o*-DCB. Bottom panels show the spectra measured in thin films prepared from solutions described above for (d) P3HT/TCNQ/BCF, (e) MeLPPP/F4TCNQ/BCF, and (f) F8BT/F6TCNNQ/BCF. All spectra were measured without air exposure using special setups, as described in the Experimental Methods section. The spectra are normalized to the absorption of neutral polymer chains in each case. The dashed vertical lines indicate the expected positions for the absorption of F*x*TCN(N)Q anions.

Figure 2 shows the optical absorption spectra of solutions and thin films of P3HT, MeLPPP, and F8BT doped with TCNQ, F4TCNQ, and F6TCNNQ, respectively, and by the BCF-coordinated variants TCNQ-4(BCF), F4TCNQ-4(BCF), and F6TCNNQ-4(BCF), respectively. For comparison, the spectra for BCF-doped polymers at a dopant ratio of 4:10 are also included. The dopant ratio (D/H) is defined as the number of dopant molecules “D” per number of host monomer units “H” mixed in solution. BCF-coordinated TCNQ-derivatives exhibit similar optical absorption spectra in solution to those of the parent TCNQ-derivative, as shown in Figure S1, with the exception of F6TCNNQ-4(BCF), which shows an additional feature at ∼3.5 eV. The unchanged low-energy absorption features indicate that bare F*x*TCN(N)Qs and BCF-coordinated F*x*TCN(N)Qs in the neutral state possess the same conjugated electronic systems.

As-prepared P3HT solution in CHCl_3_ (black line in Figure 2a) exhibits the spectrum of well-dissolved P3HT, with a prominent broad peak at ∼2.7 eV ascribed to nonaggregate and neutral P3HT chains.^40–42^ TCNQ-doped P3HT (1:10) in solution (red line in Figure 2a) shows a similar spectrum to the as-prepared P3HT with an additional feature at ∼3.1 eV, which is ascribed to neutral TCNQ molecules.^43^ The absence of TCNQ anions signatures expected at ∼1.45 and ∼1.6 eV (indicated by the positions of the vertical dashed lines in Figure 2a)^43,44^ and the abundance of neutral TCNQ in the spectrum indicate that ICT between P3HT and TCNQ does not occur as predicted by the energetically unfavorable offset between EA_TCNQ_ and IE_P3HT_.^45^ Doping P3HT with TCNQ-4(BCF) in solution with a dopant ratio of 1:30 (green line in Figure 2a) results in a spectrum resembling doped P3HT; this is characterized by the appearance of (i) positive polaron (positively charged polymer chain segment) features of aggregated chains, *i.e.*, P2_a_ at ∼1.3 eV, P2_b_ at ∼1.65 eV, and P1 < 0.6 eV, as well as (ii) signatures of aggregated neutral P3HT chains at ∼2.2 and ∼2.0 eV due to the decreased solubility of doped P3HT.^40^ We note that the increase in the intensity of the polaron-related absorption is not accompanied by the appearance of signatures of TCNQ anions, indicating that the TCNQ-4(BCF) dopant has a different electron density rearrangement upon charging compared to TCNQ alone. To rule out the possibility that the increased doping efficiency observed for TCNQ-4(BCF) over TCNQ is due to simple doping by BCF, which is known to dope P3HT,^8^ we show the spectrum of BCF-doped P3HT in solution (blue line in Figure 2a) at a dopant ratio of 4:10. The spectrum of BCF:P3HT is similar to that of TCNQ-4(BCF):P3HT (blue and green lines in Figure 2a); however, it later shows a 3-fold higher intensity of the P3HT-polaron-related absorption. The absence of TCNQ anions signatures in the absorption spectrum of TCNQ-4(BCF) and the higher intensity of polaron-related signatures (as compared to BCF:P3HT) indicate that the observed doping effect is not due to TCNQ or BCF alone but is indeed caused by the BCF-coordinated TCNQ molecules.^39^

Next, we discuss the doping of MeLPPP (IE ≈ 5.4 eV) with F4TCNQ, F4TCNQ-4(BCF), and BCF. The optical absorption spectrum of as-prepared MeLPPP in *o*-DCB (black line in Figure 2b) shows a well-resolved vibronic structure of the fundamental absorption of neutral MeLPPP due to the planarized backbone.^15,46,47^ Three peaks are observed at ∼2.7, 2.9, and ∼3.1 eV. F4TCNQ:MeLPPP (1:10) in *o*-DCB (red line in Figure 2b) shows a similar spectrum to that of as-prepared MeLPPP with no signs of doping. The absence of the polaron-related absorption features of MeLPPP (reported to appear at ∼1.9 and ∼0.4 eV)^15^ and F4TCNQ anion absorption features expected at ∼1.45 and ∼1.6 eV (indicated by the position of the dashed lines in Figure 2b)^6,43^ corroborate that F4TCNQ does not undergo charge transfer with MeLPPP as predicted by the energy offset between EA_F4TCNQ_ and IE_MeLPPP_. Once F4TCNQ-4(BCF) is used as a dopant for MeLPPP in solution at a ratio of 1:10, clear polaron-related absorption features at ∼1.9 and <0.6 eV appear with a significant intensity, as shown by the green line in Figure 2b, without any signatures from F4TCNQ anions.^15^ The absorption spectrum of BCF:MeLPPP (4:10) in *o*-DCB (green line in Figure 2b) shows negligible doping. The significantly higher doping efficiency observed for F4TCNQ-4(BCF):MeLPPP as compared to F4TCNQ:MeLPPP and BCF:MeLPPP—qualitatively estimated by comparing the intensity of polaron-related absorption features to the intensity of neutral polymer absorption features—is again attributed to the increased oxidation strength of F4TCNQ-4(BCF).

To further underpin the potency of our approach, we discuss doping of F8BT, which has the highest IE ≈ 5.9 eV of the polymers investigated here. The optical absorption spectra of F8BT doped with F6TCNNQ, F6TCNNQ-4(BCF), and BCF in solution (*o*-DCB) are shown in Figure 2c. As-prepared F8BT shows a broad feature at ∼2.7 eV ascribed to neutral F8BT chains (black line in Figure 2c).^15,48^ Upon doping with F6TCNNQ with a dopant ratio of 1:10, polaron-related absorption features are absent (red line in Figure 2c), indicating that doping does not occur as previously reported and also expected from the energy offset of EA_F6TCNNQ_ and IE_F8BT_.^15^ Once F6TCNNQ-4(BCF) is used as the dopant (dopant ratio of 1:10), clear polaron-related absorption is visible at ∼1.7 and <0.6 eV (green line in Figure 2c).^15,48^ Similar to the cases above, the expected absorption of F6TCNNQ anions at ∼1.27 and ∼1.08 eV (indicated by the positions of the vertical dashed lines in Figure 2c)^23^ is not observed. Furthermore, BCF does not induce any observable doping effect in F8BT at the dopant ratio of 4:10 (blue line in Figure 2c). Accordingly, the coordination of F6TCNNQ with four BCF results in a stronger p-dopant capable of doping polymers with IE as high as ca. 5.90 eV. Using F4TCNQ-4(BCF) as the dopant with F8BT results in no observable doping effect, as shown in Figure S2, indicating that the amount of the increase in the oxidation strength of BCF-coordinated F*x*TCN(N)Q is well correlated with the oxidation strength of the F*x*TCN(N)Q acceptor molecules, which agrees with DFT calculations, *vide infra*.

Absorption spectra of thin films of the aforementioned dopant:host pairs are shown in Figures 2d–f for P3HT, MeLPPP, and F8BT, respectively. The general observations and conclusions discussed for the solution spectra are fully analogous for the thin film spectra. We note that the kink observed around 1.5 eV is due to the detector change of our setup at ∼860 nm, which results in an intensity offset of the recorded signal >860 nm. The correction of this artifact was carried out by aligning the intensity of the spectra in both regions. However, the observed persistence of the kink may be related to the small thickness and limited uniformity of the films due to aggregation in solution.

It is worth noting that the observed doping efficiency for MeLPPP and F8BT doped with F4TCNQ-4(BCF) and F6TCNNQ-4(BCF), respectively, is larger than that of the strong Mes_2_B^+^[B(C_6_F_5_)_4_]^−^ p-dopant (with an EA_eff_ of 5.9 eV), which was reported previously to dope both polymers at a similar dopant ratio of 1:10 (see Figure S3).^15^ This points toward the significantly increased oxidation strength of F*x*TCN(N)Q once coordinated with BCF and the viability of the approach presented in this work to increase the window of available strong p-dopant molecules.

To further confirm the increased oxidation strength of the BCF-coordinated F*x*TCN(N)Q dopants compared to F*x*TCN(N)Q, we have performed DFT calculations (wB97XD/Def2-SVP) for neutral and monoanionic species and calculated the adiabatic EAs in the gas phase, as shown in Table 1.

**Table 1:** Calculated Adiabatic Electron Affinities (eV) in the Gas Phase for F*x*TCN(N)Q and F*x*TCN(N)Q-4(BCF)

TCNQ	TCNQ-4(BCF)	F4TCNQ	F4TCNQ-4(BCF)	F6TCNNQ	F6TCNNQ-4(BCF)
3.67	6.39	4.16	6.85	4.43	6.88

**Figure 3:**
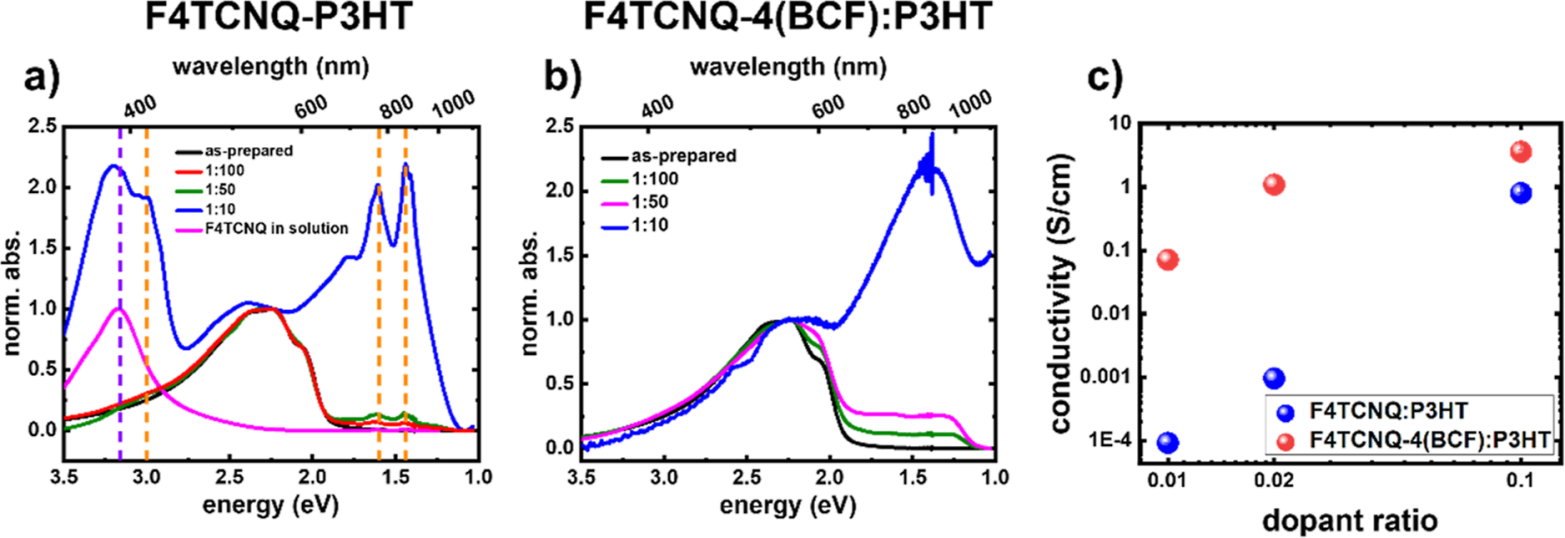
Optical absorption spectra of doped P3HT thin films as a function of the dopant ratio for the dopants (a) F4TCNQ and (b) F4TCNQ-4(BCF). (c) Electrical conductivity of doped P3HT with either F4TCNQ or F4TCNQ-4(BCF) as a function of the dopant ratio. Absorption spectra are measured in air, while conductivity measurements are measured inside an inert-gas glovebox. The vertical dashed lines in (a) indicate the position of the neutral F4TCNQ (purple) and the F4TCNQ anions (orange). We have further investigated the influence of the number of BCF molecules in the coordinated F4TCNQ, in F4TCNQ:*x*(BCF), on the degree of doping of P3HT, where *x* = 0, 1, 2, 3, and 4, as well as BCF-doped P3HT for comparison, as shown in Figure 4. Increasing the number of BCF molecules in the coordinated F4TCNQ increases the degree of doping is qualitatively evident from the intensity ratio between the neutral P3HT absorption peak and the polaron-related absorption peak. Furthermore, the F4TCNQ anions are visible in the spectra up to *x* = 2. At *x* = 3 there is no visible signature for F4TCNQ anions, indicating that most of the F4TCNQ molecules in the solution are coordinated with BCF. Some unreacted F4TCNQ can still be observed in the spectra (at ∼3.2 eV), which indicates that instead of forming completely coordinated F4TCNQ-3(BCF), we have F4TCNQ-4(BCF) and uncoordinated F4TCNQ. At *x* = 4, complete bleaching of the neutral P3HT peak is observed, with no indication of any F4TCNQ neutral molecules or anions.

Having established the increased oxidation strength of BCF-coordinated F*x*TCN(N)Q, we compared the electrical transport properties of a polymer doped with either F*x*TCN(N)Q or F*x*TCN(N)Q-4(BCF). In order to establish a reliable comparison, we chose the prototypical F4TCNQ:P3HT pair and compared it to F4TCNQ-4(BCF):P3HT since F4TCNQ readily dopes P3HT even without BCF coordination.^6^Figure 3a,b shows the optical absorption spectra of F4TCNQ:P3HT and F4TCNQ-4(BCF):P3HT with increasing dopant ratios, respectively. In both cases, the increased dopant ratio results in a progressive increase in the intensity of the polaron-related absorption features and bleaching of the neutral P3HT signal, to which the spectra are normalized. The spectra of neutral F4TCNQ in *o*-DCB are shown in Figure 3a, along with guiding dashed lines at the positions of the absorption features of neutral and anion F4TCNQ. Neutral F4TCNQ exhibits a single absorption feature at around 3.16 eV (purple vertical line), while F4TCNQ anions exhibit three peaks, namely, 3.00, 1.60, and 1.44 eV.^43^ As shown in Figure 3a, increasing the amount of F4TCNQ in P3HT increases the intensity of the absorption features of the F4TCNQ anions. At dopant ratios below 1:10, there is no strong absorption of the neutral F4TCNQ, indicating that the available F4TCNQ in the solution has mostly undergone ICT with P3HT and is present in the anionic form. The abundance of the absorption feature of F4TCNQ at the dopant ratio of 1:10 indicates that not all F4TCNQ in the mixture has undergone ICT with P3HT. We note that in the case of F4TCNQ-4(BCF):P3HT no signatures from F4TCNQ (neutral or negatively charged) are present in the spectra, in line with the observation mentioned above that the BCF-coordinated dopant has a different electron density rearrangement upon charging compared to F4TCNQ alone.

By comparing Figure 3a,b, it can be seen that for the lower dopant ratios (1:100 and 1:50) F4TCNQ-4(BCF):P3HT results in higher doping efficiency as compared to F4TCNQ:P3HT at a similar dopant ratio. More explicitly, the relative intensity of the polaron-related features with respect to the neutral P3HT absorption for F4TCNQ-4(BCF):P3HT at a 1:100 dopant ratio matches that for F4TCNQ:P3HT at a dopant ratio of 1:50. This implies that the increase in the oxidation strength for BCF-coordinated F4TCNQ discussed above increases the driving force for forming polarons.^49^ At the dopant ratio of 1:10 for both dopants, the intensity of the neutral P3HT chain absorption significantly bleaches with yet higher polaron-related absorption in F4TCNQ-4(BCF):P3HT.

**Figure 4:**
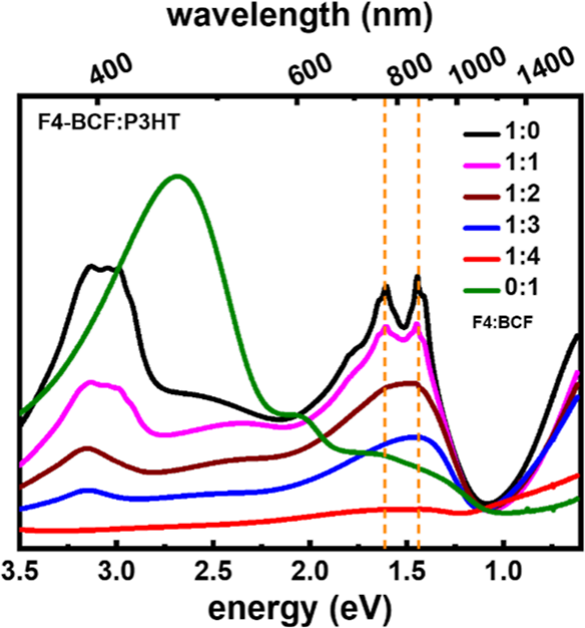
Optical absorption spectra of doped P3HT with F4TCNQ-*x*(BCF), *x* = 0–4, and BCF at a molar dopant ratio of 1:10. Spectra are measured in solution (*o*-DCB) without air exposure. The vertical dashed lines (orange) are at the position of F4TCNQ anions absorption.

The electrical conductivity of P3HT doped with either dopant as a function of the dopant ratio is shown in Figure 3c. In line with the optical absorption data for both dopants, the electrical conductivity increases with an increase in the dopant ratio. We observe that for F4TCNQ-4(BCF):P3HT, the electrical conductivity is higher than that for F4TCNQ:P3HT for all dopant ratios. At a dopant ratio of 1:100 for F4TCNQ-4(BCF):P3HT, the electrical conductivity is larger by 3 orders of magnitude as compared to that of F4TCNQ:P3HT at the same dopant ratio and 2 orders of magnitude larger than that of 1:50 F4TCNQ:P3HT, which has a similar ionization efficiency as inferred from the optical absorption spectra. This indicates that in addition to the higher ionization efficiency, BCF-coordinated F4TCNQ is also capable of generating a higher density of mobile charge carriers in P3HT, *i.e.*, it exhibits a higher doping efficiency. The conductivity at 1:50 dopant ratio of F4TCNQ-4(BCF):P3HT matches that of F4TCNQ:P3HT at 1:10 dopant ratio. This lends further support to the notion that BCF-coordinated F4TCNQ exhibits a higher doping efficiency for P3HT than that of F4TCNQ alone. The higher doping efficiency of F4TCNQ-4(BCF):P3HT may be attributed to reduced electrostatic interaction between the dopant anion and the positive polarons formed on the P3HT chains due to the larger spatial separation between the core of the dopant anion and the polymer chains, allowing for the formation of delocalized polarons. This is analogous to the earlier report on using shielded dodecaborane-based molecules as a p-dopant for P3HT, which has resulted in ∼100% doping efficiency (Figure 4).^49,50^

Comparing the electrical conductivity of F4TCNQ-4(BCF):P3HT to F4TCNQ:P3HT at the dopant ratio 1:10 shows that the increase in conductivity is not as substantial as compared to the case of lower dopant ratios (1:100 and 1:50). This can be explained by the fact that changes in electrical conductivity in doped polymers result from both changes in the density of free charge carriers and the mobility of the film. Previously reported trends for electrical conductivity *vs* dopant ratio show an initial increase of conductivity due to the increase of the amount of free charge carriers in the film, which compensates for any decrease in mobility due to Coulomb scattering effects from the dopant anions. The conductivity continues to increase with increasing the dopant ratio up to a maximum value, after which the decrease in mobility due to scattering and disrupting the crystallinity and morphology of the films dominates, and the conductivity decreases with further increases in the dopant ratio.^5,15^ Thus, we anticipate that F4TCNQ-4(BCF) and F4TCNQ influence the changes in charge mobility in P3HY differently due to the different sizes of the dopants. The larger dopant F4TCNQ-4(BCF) can be expected to disrupt the morphology of the films to a greater extent and possibly have reached the maximum conductivity earlier as compared to F4TCNQ alone.

The anticipated three-dimensional structure of the BCF-coordinated F4TCNQ dopant and its larger size as compared to the parent two-dimensional planar F4TCNQ motivated us to evaluate the dopant stability in P3HT against drift under an externally applied electric field. In the ICT p-doping mechanism, the charge transfer from the polymer host to the neutral dopant molecule results in a positive polaron on the polymer and a negatively charged dopant molecule that acts as a counteranion to maintain charge neutrality. The anions are influenced by external electric fields and can drift in a direction opposite to the hole current flow under device operation conditions. In general, dopant diffusion in polymer hosts has been observed to decrease with increased size of the dopant molecules and for molecules with a bulky structure.^28,34^ In the following, we demonstrate that F4TCNQ-4(BCF) indeed undergoes less drift under externally applied electric fields compared to F4TCNQ.

**Figure 5:**
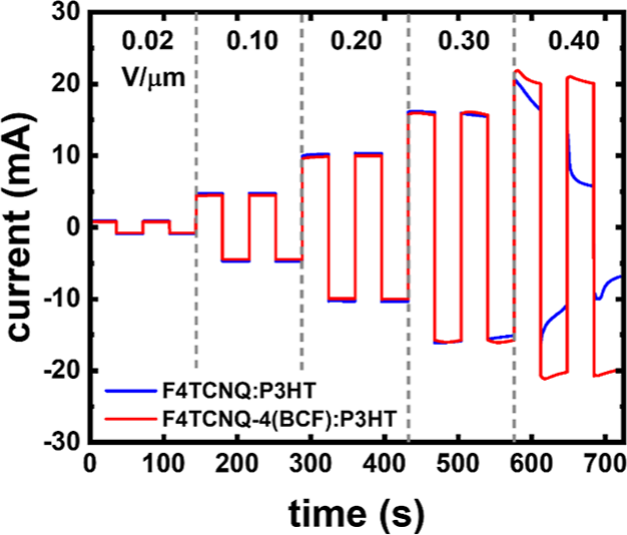
Current as a function of time under an applied electric field with reversed polarity for thin films of F4TCNQ:P3HT (1:10) and F4TCNQ-4(BCF):P3HT (1:50). The applied electric field (values given on top of the figure) was increased over certain periods of time, as indicated by the vertical dashed lines (details in the Experimental Methods section).

In Figure 5, we compare the changes in the current as a function of time for increasing steps of applied constant electric field cycles of reversed polarity in F4TCNQ:P3HT (1:10) and F4TCNQ-4(BCF):P3HT (1:50), following the protocol set out by Müller *et al.*(29) (detailed in the Experimental Methods section). To reduce the effect of resistive sample heating (*I*^2^*R*, where *I* is the electrical current passing through the sample and *R* is the resistance of the sample) and accordingly eliminate the influence of potential thermal dedoping on the comparison, dopant ratios of F4TCNQ:P3HT at 1:10 (*R* ∼ 950 Ω) and F4TCNQ-4(BCF) at 1:50 (*R* ∼ 1070 Ω) are selected for comparison as they show similar levels of electrical current (see Supporting Information Figure S4 for comparison of other dopant ratios). For each cycle of constant electric field (separated by the vertical dashed lines in Figure 5), the polarity of the field is reversed twice, corresponding to the periodic reversal of the direction of the current (*y*-axis). The reversed polarity laterally sweeps mobile dopant anions in cycles from the negative electrode to the positive electrode, passing through the uniform distribution of the dopant in the host material upon switching the polarity. At low applied electric fields (<0.3 V/μm), the current is essentially constant with time for both dopants, indicating no changes in the dopant lateral distribution in P3HT. A different behavior is observed starting at an electric field of 0.3 V/μm, at which the current clearly decreases with time for F4TCNQ:P3HT, indicating drift of the dopant anions, which results in a dedoped region close to the negative electrode. This, in turn, increases the overall resistivity of the film and results in the observed decrease in the current. In contrast, negligible changes are observed in the case of F4TCNQ-4(BCF):P3HT up to an electric field of 0.3 V/μm. At higher applied electric fields of 0.4 V/μm, both F4TCNQ and F4TCNQ-4(BCF) dopants appear to drift as inferred by the decrease of the current; however, a larger decrease in the case of the F4TCNQ is observed, which confirms the higher dopant stability against drifting under externally applied electric fields for F4TCNQ-4(BCF). Furthermore, in the case of F4TCNQ:P3HT, the value of the current is not reversible with the cycling of the polarity at 0.4 V/μm. This indicates that some dopant anions are rendered inactive (causing an overall dedoping) rather than sweeping spatially between the electrodes. We speculate that the loss of dopants in the film results from reaction at the positive electrode and/or dopant out-diffusion due to increased sample temperature driven by resistive heating. On the contrary, F4TCNQ-4(BCF):P3HT shows a nearly reversible behavior under the same conditions, pointing toward a higher stability against thermal diffusion of F4TCNQ-4(BCF).

We have further compared the thermal stability of doped P3HT with either F4TCNQ or F4TCNQ-4(BCF) by using optical absorption spectra, as shown in Figure S5. The optical absorption spectra of the doped samples were measured without air exposure and then heated inside the glovebox at 75 °C for 10 min. The optical absorption spectra of doped samples after heating were measured again without exposing them to air.

As shown in Figure S5a, the intensity of the polaron-related absorption in F4TCNQ:P3HT (1:10) decreases significantly after heating, indicating that the dopant diffuses out of the sample (dedoping) in agreement with earlier experiments.^52^ In contrast, the degree of dedoping as judged by the decrease of the polaron-related absorption after thermal annealing is smaller in the case of F4TCNQ-4(BCF):P3HT (1:10) (Figure S5b) as compared to the former case. This indicates that the doping of P3HT with the BCF-coordinated F4TCNQ has greater thermal stability, which can be attributed to the larger molecular size of the dopant.

For the evaluation of the air stability of the F4TCNQ-4(BCF) doped P3HT, we compared the optical absorption spectra of thin films with a dopant ratio of 1:10 without exposure to air to the case with air exposure. The results are shown in Figure S6. We observe that the degree of doping (as judged by the ratio of the polaron-related absorption peak to the neutral P3HT peak) increases after air exposure, in addition to changes in the shape of the polaron-related absorption peak. While these changes may indicate changes in the doped species after air exposure, the increase in the doping degree after air exposure is counterintuitive to the observation that the dopant solutions of coordinated F*x*TCN(N)Q-4(BCF) are unstable in the presence of a small amount of moisture (even inside the glovebox) due to the hygroscopic nature of BCF. We point out that the dopant solution changed color after one week of storage inside the glovebox, in agreement with a recent report on F4TCNQ-4(BCF), which was published during the revision of our present contribution, where the presence of water resulted in the formation of BCF complexes that prevent the formation of Lewis-paired p-dopants.^53^

## Conclusions

In conclusion, the increase of the oxidation strength of TCNQ, F4TCNQ, and F6TCNNQ by coordination with four BCF molecules using a simple one-step solution-mixed approach has been demonstrated. The F*x*TCN(N)Q-4(BCF) dopants are shown to efficiently ionize the polymer hosts with an IE up to 5.9 eV, which is not possible to dope with any of the F*x*TCN(N)Q molecules alone. We expect that the adduct formation between BCF and the –CN groups in F*x*TCN(N)Q molecules results in an electron-poor core of the dopants, in analogy to the process of increasing the oxidation strength of TCNQ by fluorination. The increased size of F4TCNQ-4(BCF) additionally offers a higher doping efficiency as deduced from the higher levels of electrical conductivities in doped P3HT as compared to F4TCNQ-doped P3HT at a similar dopant ratio. Anticipated disruptions in film morphology with the use of larger molecular dopants seem to have a negligible effect on the electrical transport at the used dopant ratios in this study. Furthermore, an increase in the dopant stability against drift under applied electric fields has been observed for F4TCNQ-4(BCF) in P3HT, which is beneficial for achieving highly performing organic optoelectronic devices. Our work increases the window of available strong molecular p-dopants that are capable of doping polymer hosts with higher ionization energy by simple mixing of solutions or readily available and accessible molecular dopants.

## Experimental Methods

### Sample Preparation

Poly(3-hexylthiophene-2,5-diyl) “P3HT”, weight average molecular weight (*M*_w_) of 50–100 kg mol^–1^, regioregularity >90%, was purchased from Sigma-Aldrich GmbH. MeLPPP with a *M*_w_ of 82 kg mol^–1^ was synthesized, as described elsewhere.^51^ Poly[(9,9-dioctylfluorenyl-2,7-diyl)-*co*-(1,4-benzo-{2,1′,3]thiadiazole)] “F8BT” *M*_w_ of 10 kg mol^–1^ was purchased from H.W. Sands Corp. TCNQ (product number: T2313), F4TCNQ (product number: T1131), and BCF (product number: T0078) were obtained from TCI Deutschland GmbH. F6TCNNQ was obtained from Novaled GmbH. All materials were used as-received.

The solutions of the polymers and dopant molecules were prepared inside a nitrogen glovebox (O_2_ < 0.1 ppm and H_2_O < 0.1 ppm) using either chloroform (CHCl_3_) or 1,2-dichlorobenzene (*o*-DCB)—purchased as anhydrous solvents from Sigma-Aldrich GmbH (>99.9% purity, inhibitor-free).

Stock solutions of P3HT “60.1 mM” (in either CHCl_3_ or *o*-DCB), MeLPPP “11.5 mM” (in *o*-DCB), F8BT “19.1 mM” (in *o*-DCB), and BCF “19.5 mM” (in either CHCl_3_ or *o*-DCB) were prepared at a weight concentration of 10 mg/mL. TCNQ “4.9 mM” (in CHCl_3_), F4TCNQ “3.6 mM” (in *o*-DCB), and F6TNNQ “2.8 mM” (in *o*-DCB) were prepared at a weight concentration of 1 mg/mL due to their low solubility.

Doped polymer solutions were prepared by solution-mixing of specific volumes of the stock polymer host (H) and dopant (D) solutions to yield the desired dopant ratio (D/H). The dopant ratio (D/H) is defined as the ratio between the dopant molecules per monomer unit of the polymer in solution.

BCF-coordinated F*x*TCN(N)Q (*x* = 0, 4, and 6) solutions were prepared by mixing specific volumes of the stock molecule solution to yield a ratio of four BCF molecules per one F*x*TCN(N)Q molecule, as follows:

- TCNQ-4(BCF) “2.4 mM” was prepared by mixing 0.49 mL of BCF with 0.50 mL of TCNQ solutions.
- F4TCNQ-4(BCF) “2.1 mM” was prepared by mixing 0.43 mL of BCF with 0.57 mL of F4TCNQ solutions.
- F6TCNNQ-4(BCF) “1.8 mM” was prepared by mixing 0.18 mL of BCF with 0.32 mL of F6TCNNQ solutions.

Thin films were prepared by spin-coating inside the glovebox on solvent-cleaned substrates using a spin speed of 1000 rpm for 60 s inside the glovebox. The substrates used were either glass substrates (for optical measurements) or interdigitated ITO substrates obtained from Ossila (product number S161).

### Optical Absorption Spectroscopy

A PerkinElmer Lambda950 UV–vis–NIR spectrophotometer was used to collect the optical absorption spectra in dual beam mode. The spectra were corrected with a 100% transmission. Data were measured with respect to the solvent (in the case of measurements on solution) or to the glass substrate (in the case of thin film measurements). Measured solutions were diluted by 10× to increase the measured transmission of the samples and obtain a reliable absorption signal. All measurements were collected without exposure to air using special airtight cuvettes (solutions) or nitrogen-filled boxes (thin films), unless mentioned otherwise.

### Conductivity Measurements

The *I*–*V* measurements were performed at room temperature in a nitrogen-filled glovebox. The prepatterned substrates, purchased from Ossila, had a channel width of 50 μm between ITO electrodes. For the square wave *I*–*V* measurements, the source voltage was fixed for 30 s before the voltage polarity was flipped. The current was measured every 0.1 s, with the measurement cycle repeated twice for each step in voltage.

## Supporting Information Available

Optical micrographs of solutions, additional absorption spectra, and additional transport measurements (PDF)
