# Supplementary material for: Coordination of Tetracyanoquinodimethane-Derivatives with Tris(pentafluorophenyl)borane Provides Stronger p-Dopants with Enhanced Stability
Source: ACS Appl Mater Interfaces. 2023 Sep 20;15(39):46148–56. doi: 10.1021/acsami.3c10373 (PMC10561139; doi:10.1021/acsami.3c10373)
Supplement: Supplementary file 1 — am3c10373_si_001.pdf [file am3c10373_si_001.pdf]

Supporting Information

Coordination of tetracyanoquinodimethane-derivatives with tris(pentafluorophenyl)borane provides stronger p-dopants with enhanced stability

Ahmed E. Mansour[1,2], Ross Warren[2], Dominique Lungwitz[2], Michael Forster[3], Ullrich Scherf[3], Andreas Opitz[2], Moritz Malischewski[4], Norbert Koch[1,2*]

[1] Helmholtz-Zentrum Berlin für Materialien und Energie GmbH, 12489 Berlin, Germany
[2] Institut für Physik & IRIS Adlershof, Humboldt-Universität zu Berlin, 12489 Berlin, Germany
[3] Makromolekulare Chemie and Wuppertal Center for Smart Materials and Systems (CM@S), Bergische Universität Wuppertal, 42097 Wuppertal, Germany
[4] Freie Universität Berlin, Institute of Chemistry and Biochemistry, 14195 Berlin, Germany

* corresponding author email address: norbert.koch@physik.hu-berlin.de

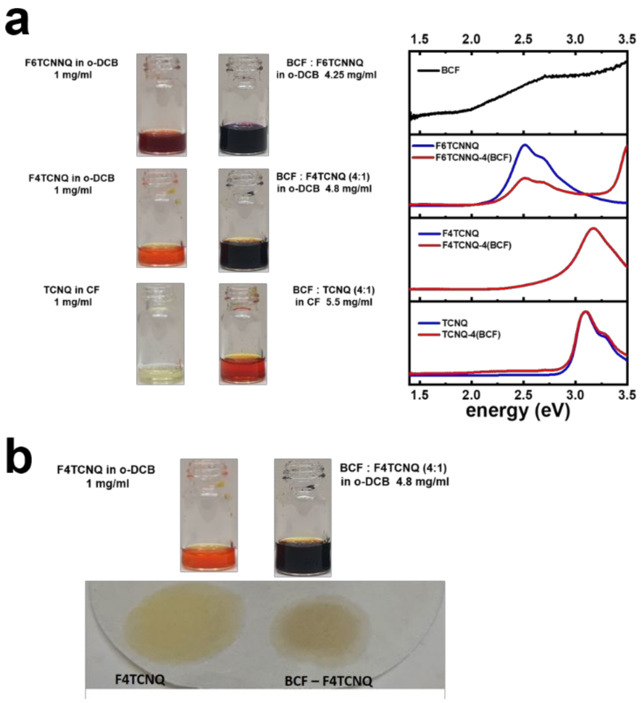

Figure S1: (a) Optical micrographs of the dopants solutions before and after coordination with four BCF molecuels, and the corresponding optical absorption spectra normalized to the maximum

absorption. The change in the color of solution is due to aggregation, which is further demonstrate in (b).

We note that the dopants before and after mixing with BCF exhibit a similar optical absorption spectra. An exception was for F6TCNNQ as compared to F6TCNNQ-4BCF which exhibited an additional feature at 3.5 eV. The origin of this feature is not clear at this stage and worth further investigation in the future.

To understand the similar absorption spectra for differently colored solution, we have dropped the solution of F4TCNQ-4BCF (as an example) on a filter paper and compared it to that of F4TCNQ as shown in Figure S1b. The filter paper has shown two color contrasts indicating the co-existence of two phases in solution for the coordinated dopant, i.e. solubilized dopants and large dopant aggregates in the solution.

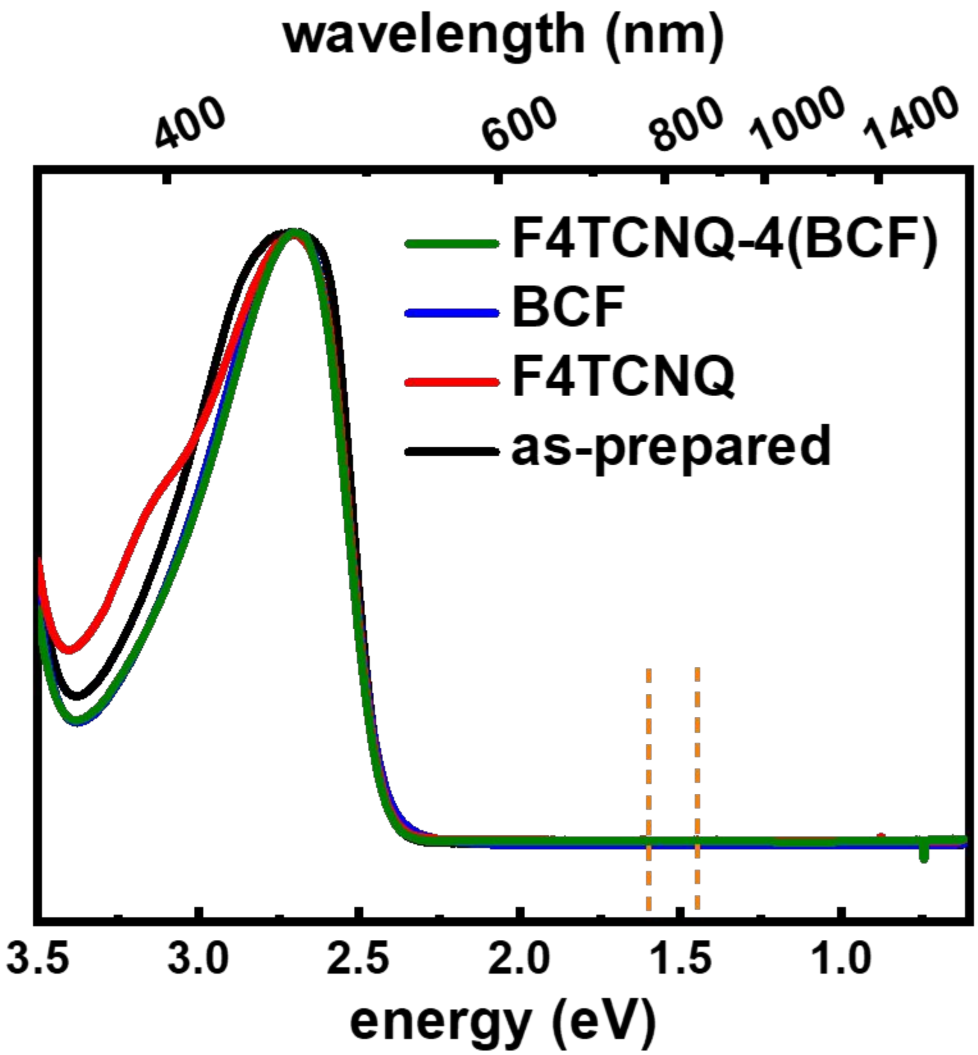

Figure S2: Optical absorption spectra in o-DCB solutions of as-prepared F8BT, F4TCNQ:F8BT (1:10), BCF:F8BT (4:10), and F4TCNQ-4(BCF):F8BT (1:10). The dashed vertical lines indicate the expected position for the absorption of F4TCNQ anions.

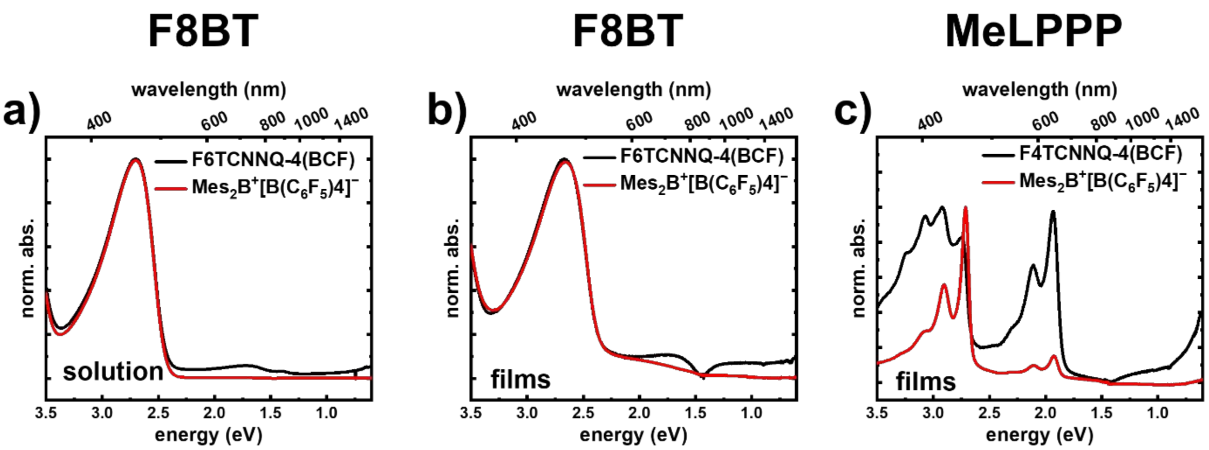

Figure S3: Comparing the optical absorption spectra of (a) doped F8BT solution in o-DCB, (b) doped F8BT thin films, and (c) doped MeLPPP thin films, using BCF coordinated TCNQ-derivative dopants (black) and $Mes_2B^+[B(C_6F_5)_4]^-$ (red). All samples are prepared with the respective dopants at a ratio of 1:10.

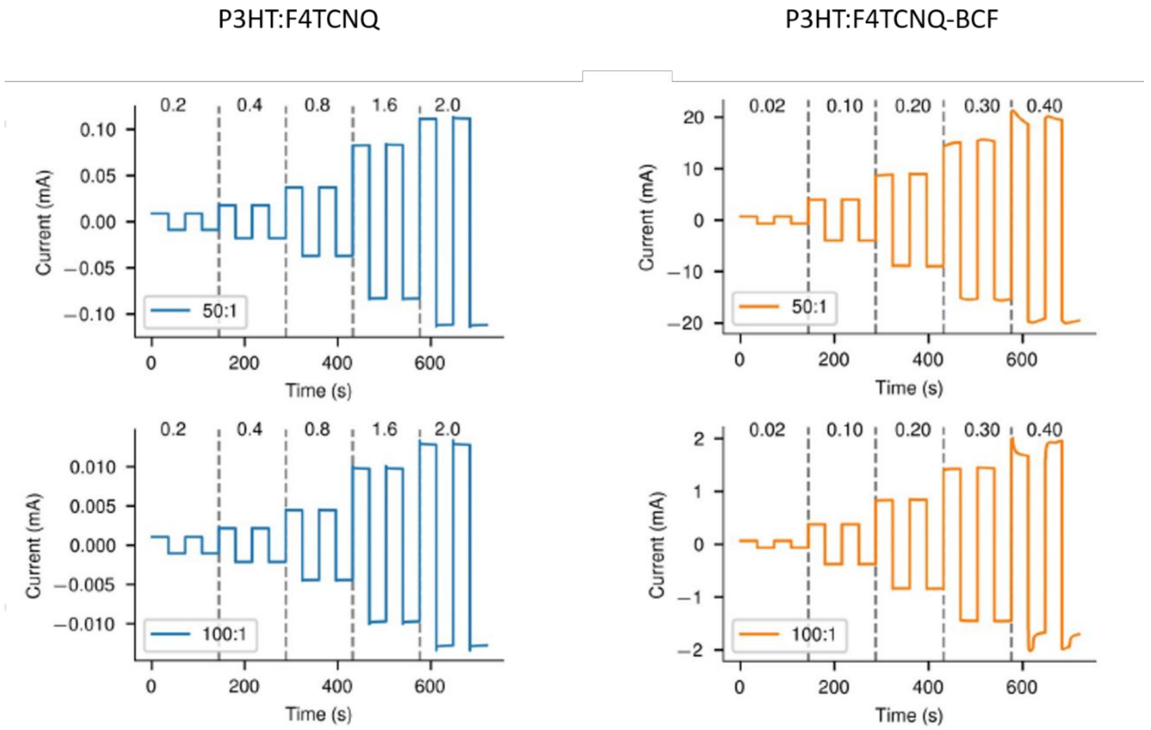

Figure S4: Current as function of time under applied electric field with reversed polarity for thin films of: F4TCNQ:P3HT (1:100 and 1:50) and F4TCNQ-4(BCF):P3HT (1:100 and 1:50). The applied electric field (values given on top of the Figure) was increased over certain periods of time as indicated by the vertical dashed lines (details in the experimental section).

Comparing the bias stability of samples of F4TCNQ:P3HT and F4-4(BCF):P3HT at similar dopant ratios, we noticed that due to the low conductivity of the former, no diffusion was observed in the former for both 1:100 and 1:50 dopant ratios, when compared to the more conductive F4-BCF:P3HT. A conclusion on bias stability couldn't be made due to the different level of currents in both samples, and thus different Joule heating effect (P=I2R). In order eliminate the influence of the Joule heating in our comparison, we chose samples having a similar level of current. For this reason, F4TCNQ:P3HT at a dopant ratio of 1:10 (R = 950 Ohms) was compared to F4TCNQ-4(BCF):P3HT (R = 1070 Ohms) at a dopant ratio 1:50, as shown overlayed (without normalization) in Figure 5.

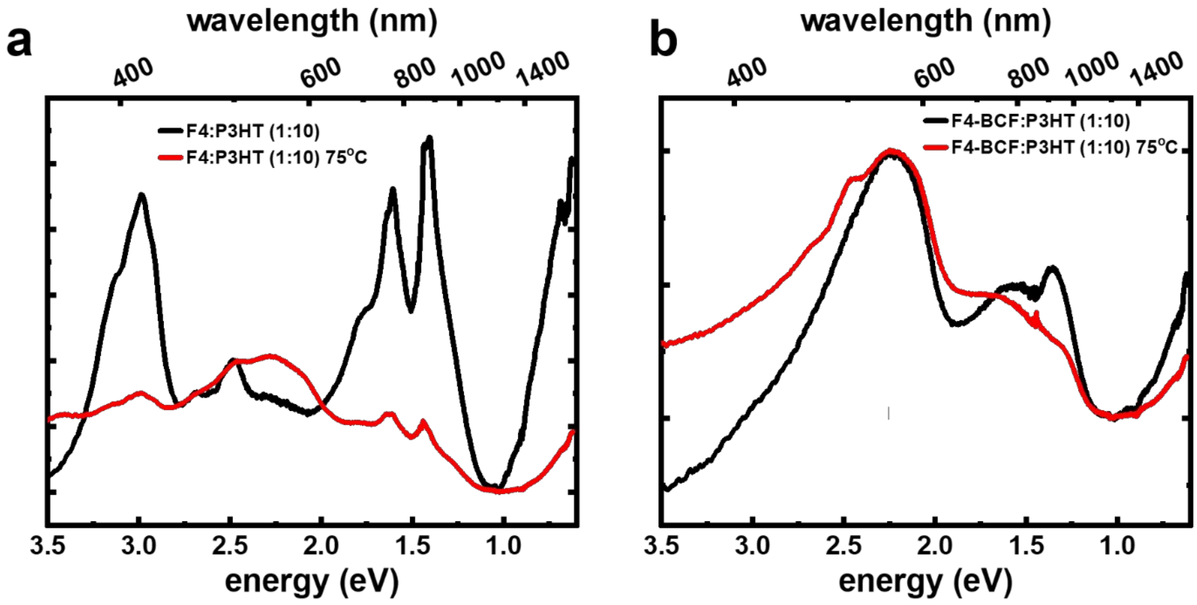

Figure S5: Optical absorption spectra of doped P3HT before and after heating at 75 °C for 10 minutes inside the glovebox for (a) F4TCNQ:P3HT (1:10) and (b) F4TCNQ-4(BCF):P3HT (1:10). The measurements were performed without air exposure. The spectra are normalized the neutral P3HT peak.

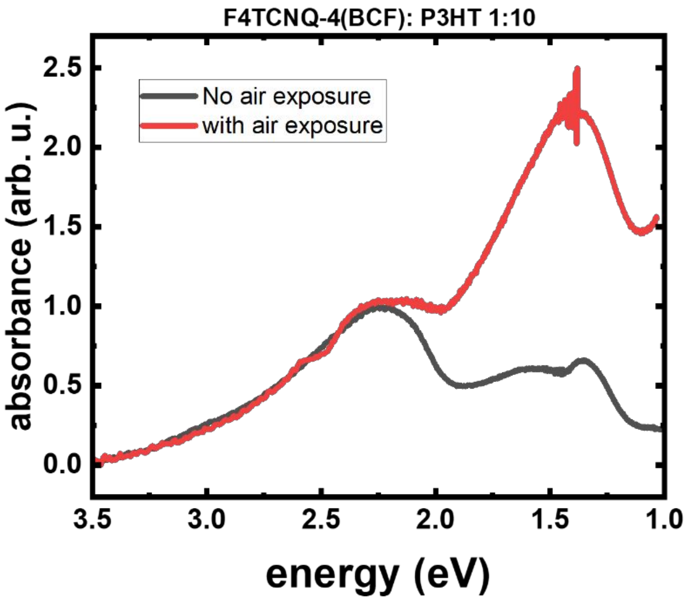

Figure S6: Optical absorption spectra of F4TCNQ-4(BCF):P3HT (1:10) measured without air exposure (black) and in air (red). Spectra are normalized to the neutral P3HT peak.
